# Barriers to increasing paid parental leave in U.S. neurology residencies: a survey of program directors

**DOI:** 10.1186/s12909-024-05333-1

**Published:** 2024-04-09

**Authors:** Sarah E. Conway, Wei Wang, Sashank Prasad

**Affiliations:** 1 Department of Neurology, Brigham and Women’s Hospital, 60 Fenwood Road, Boston, MA USA; 2 Harvard Medical School, Boston, MA USA; 3 Departments of Medicine and Neurology, Brigham and Women’s Hospital, Boston, MA USA; 4 Division of Sleep Medicine and Department of Medicine, Harvard Medical School, Boston, MA USA; 5 Department of Neurology, University of Pennsylvania Perelman School of Medicine, Philadelphia, PA USA

**Keywords:** Parental leave, Graduate medical education, Neurology residency

## Abstract

**Background:**

The American Board of Psychiatry and Neurology (ABPN) and the Accreditation Council for Graduate Medical Education (ACGME) require that residency programs allow at least 6 weeks of parental leave. The American Medical Association (AMA) recommends 12 weeks of paid parental leave. Despite these recommendations, there is little information about parental leave policies across U.S. neurology residencies. The objective of our study was to assess parental leave policies in U.S. adult neurology residencies and barriers to increasing the duration of leave.

**Methods:**

We distributed an anonymous online survey to U.S. adult neurology program directors (PDs) to assess demographics, components and length of parental leave, perceived impact on residents’ clinical training and academic development, and barriers to increasing the length of leave.

**Results:**

We contacted 163 PDs and received 54 responses (response rate of 33%). 87% reported policies for both childbearing and non-childbearing residents. The average maximal length of leave allowed without extension of training was 8.5 weeks (range 0–13) for childbearing and 6.2 weeks (range 0–13) for non-childbearing residents. Most PDs felt that parental leave had a positive impact on resident wellness and neutral impact on clinical competency, academic opportunities, and career development. The most common barriers to providing a 12-week paid policy were concerns about equity in the program (82%), staffing of clinical services (80%), and impact on clinical training (78%).

**Conclusions:**

Although most programs in our study have parental leave policies, there is significant variability. Policies to improve parental leave should focus on addressing common barriers, such as additional solutions to staffing clinical services.

**Supplementary Information:**

The online version contains supplementary material available at 10.1186/s12909-024-05333-1.

## Background

Parental leave is an increasingly important issue in graduate medical education. Paid parental leave has been associated with substantial health benefits for parents and children including reductions in infant mortality and numerous childhood illnesses, improvement in maternal mental health, and increases in rates of breastfeeding (the same degree of benefits are not associated with unpaid parental leave policies) [1]. 

The American Board of Psychiatry and Neurology (ABPN) recently updated its stance towards parental leave and requires that programs allow at least 6 weeks of parental leave without exhausting vacation time and nonclinical rotations and without mandatory extension of training [2]. Similarly, as of July 1, 2022, the Accreditation Council for Graduate Medical Education (ACGME) mandates that all ACGME-accredited programs provide a minimum of 6 weeks of paid parental leave [3]. A *New England Journal of Medicine* editorial in 2019 and American Medical Association (AMA) guidelines from 2022 recommend that paid leave be at least 12 weeks [4, 5]. Though it is encouraging to see these recommendations, it is unclear whether neurology residencies are consistently incorporating these guidelines in practice.

We sought to understand and describe current parental leave policies in neurology residencies and analyze perceived barriers to increasing the duration of paid leave. These important questions have not been well addressed in neurology in the way that they have been in surgical subspecialties [6–10] and medical subspecialities including psychiatry [11] and pediatrics [12, 13]. Awareness of the most relevant barriers to increasing parental leave during neurology residency is critical to achieving better policies for trainees in our field, which could also lead to improved health outcomes for children and better experiences for the physician parent.

## Methods

This study was determined to be exempt from IRB review per the Mass General Brigham institutional review board.

We created an online survey (Appendix A, supplementary material) to evaluate duration and composition of programs’ parental leave policies, program directors’ (PDs) perceptions of parental leave, perceived impact on residents, and perceived barriers to increasing duration of paid leave. Our survey was developed based on prior surveys used to evaluate parental leave policies in other medical and surgical specialities [7, 11, 14, 15]. Length of leave was measured in whole numbers from 0 to 12 weeks, with an option for greater than 12 weeks, and an option for “I’m not sure”. Childbearing leave was defined as leave for a resident who gave birth. Non-childbearing leave was defined as leave for a non-childbearing parent (either a partner of a childbearing parent, or adoption).

U.S. adult neurology PDs and their email addresses were identified via FREIDA, the American Medical Association’s online residency program database. 163 programs were contacted via email. There was no renumeration for participation. Data was collected via REDCap between 3/16/22 and 4/18/22. Survey responses were anonymous.

### Statistical analysis

Descriptive statistics such as frequency counts and percentages were calculated. Spearman correlation was estimated to determine if length of leave correlated with program size. Logistic regression models were used to assess if the perceived impact of parental leave on residents (negative impact vs. neutral or positive impact), and perceived barriers (major or minor barriers vs. no barriers) were influenced by maximum length of leave, program size, percentage of females, average number of leaves per year, and program location.

## Results

### Program demographics

**Fig. 1:**
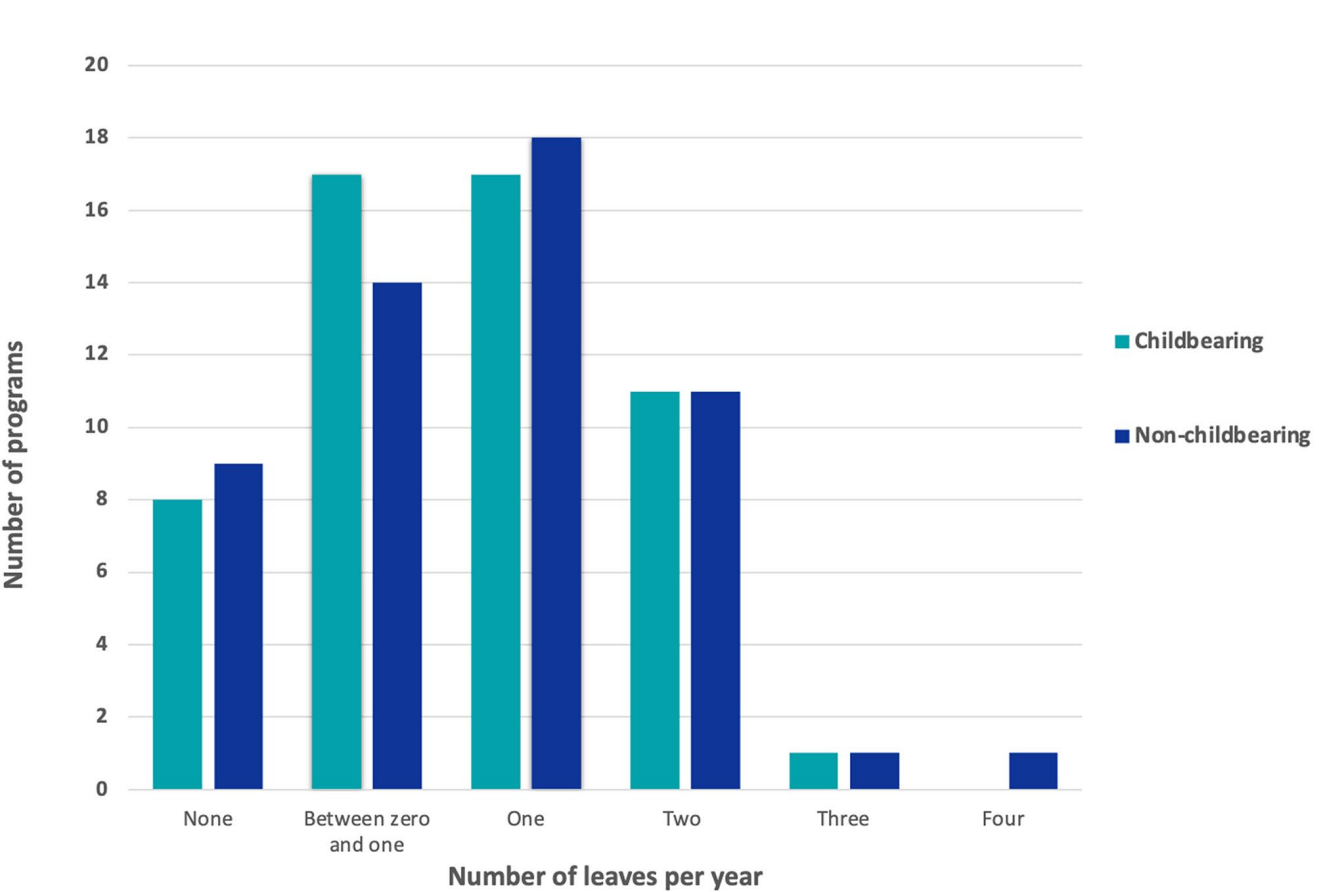
Number of parental leaves per year reported by PDs are shown on the x-axis. Number of programs is shown on the y-axis. Childbearing and non-childbearing residents are indicated by different colors

We received 54 responses, for a response rate of 33%. Most programs (50%) had between 6 and 10 residents per post-graduate year. Additional program demographics are shown in Table 1. Number of parental leaves per year are shown in Fig. 1.

**Table 1:** Program demographics

Number of adult residents per year	N (%)
1 to 5	16 (30)
6 to 10	27 (50)
11 to 15	5 (9)
16 and greater	6 (11)
**Percentage female**	
< 20%	2 (4)
20% to < 40%	8 (15)
40% to < 60%	34 (63)
60 to < 80%	10 (18)
**Location**	
Northeast	20 (37)
Midwest	13 (24)
Southeast	11 (20)
West/Southwest	10 (19)
**Years as program director**	
0 to 5 years	28 (52)
6 to 10 years	14 (26)
> 10 years	12 (22)

### Characterization of existing parental leave policies

47 (87%) programs have parental leave policies for both childbearing and non-childbearing residents, 4 (7%) programs have policies for childbearing residents only, and 3 (6%) programs have no policy or were not sure if they have a policy. Of the 47 programs with parental leave policies for both childbearing and non-childbearing residents, 30 (64%) stated the policy was the same for childbearing and non-childbearing residents, 16 (34%) stated it was different, and 1 (2%) program was not sure.

Many policies were recently updated at the time of our survey: 20 (39%) within the past year, 20 (39%) two to five years ago, 2 (4%) five to ten years ago, 2 (4%) greater than 10 years ago, and 7 (14%) programs were not sure when their policies were last updated. 13 (25%) programs were in the process of revising their parental leave policies.

PDs were asked to identify everyone involved in scheduling a leave. Responsibility for scheduling an individual leave fell upon the trainee (73%), followed by chief residents (65%), and the PDs (59%). 100% reported that other neurology residents covered for those taking a parental leave. 14% utilized attending coverage, and 4% utilized fellow coverage. Only 10% had advanced practice providers (APPs) cover for the resident taking leave. 65% offered full pay and benefits for the duration of the parental leave, 22% offered full pay and benefits for a portion of leave and reduced pay for the remainder, 6% offered reduced pay throughout the leave, and the rest were not sure.

**Fig. 2:**
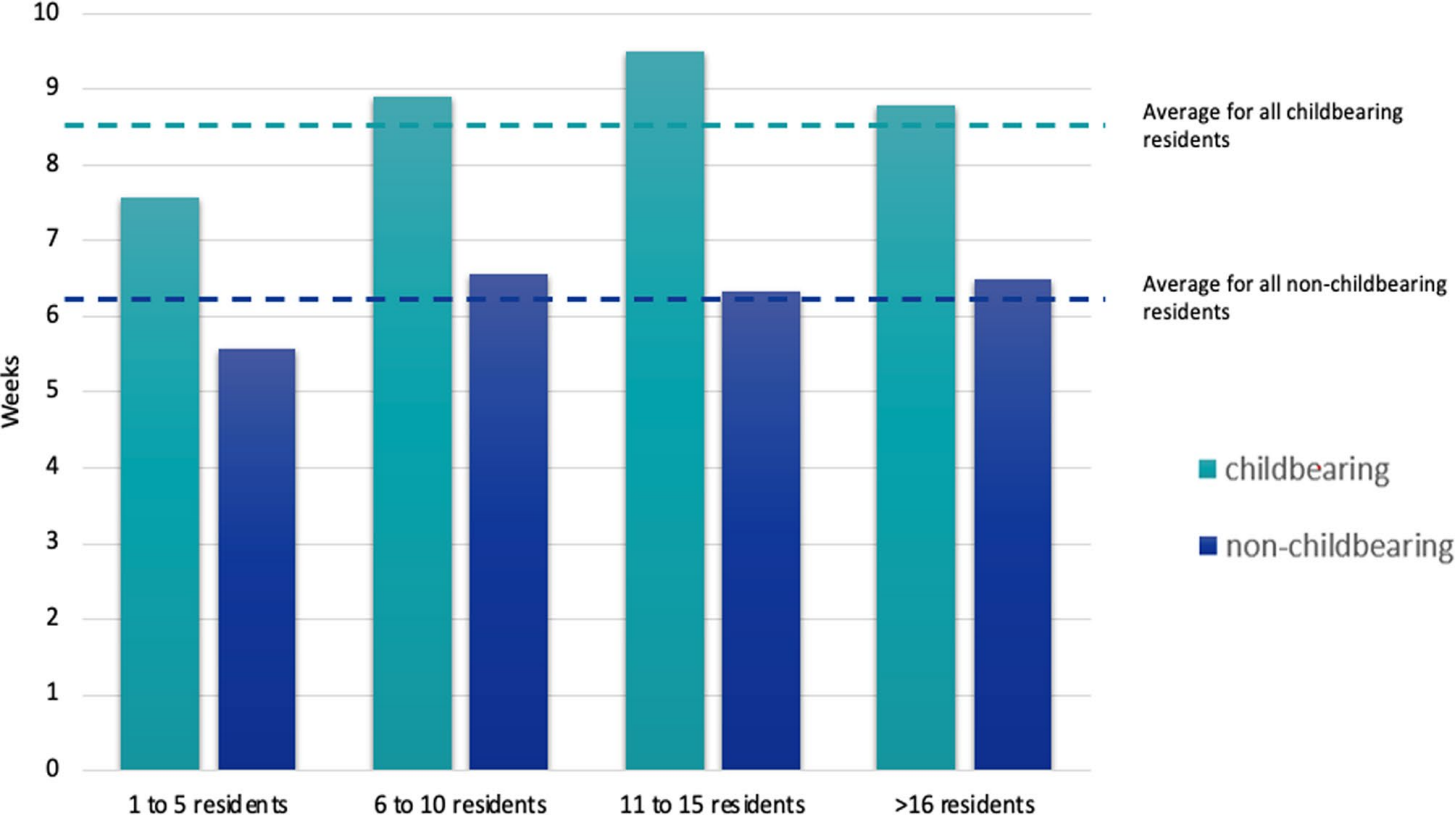
Average maximal weeks of leave are shown on the y-axis, and are stratified by residency program size on the x-axis. Childbearing and non-childbearing leave are indicated by different colors. There was no statistical correlation between program size and length of leave

The average maximal length of leave allowed without extension of training was 8.5 weeks (range 0–13) for childbearing residents and 6.2 (range 0–13) for non-childbearing residents (Fig. 2). There was no correlation between length of leave and program size (*r* = 0.20, *p* = 0.18 and *r* = 0.16, *p* = 0.31 for childbearing and non-childbearing residents respectively).

Most programs did not require full use of vacation (51%) or elective time (59%). 16% required use of all vacation time, and only 2% required use of all elective time. 42% had policies for reducing clinical rotations to create a leave. Most programs (59%) did not require residents to make up call they missed as part of their leave.

### Perceived impact of parental leave

**Fig. 3:**
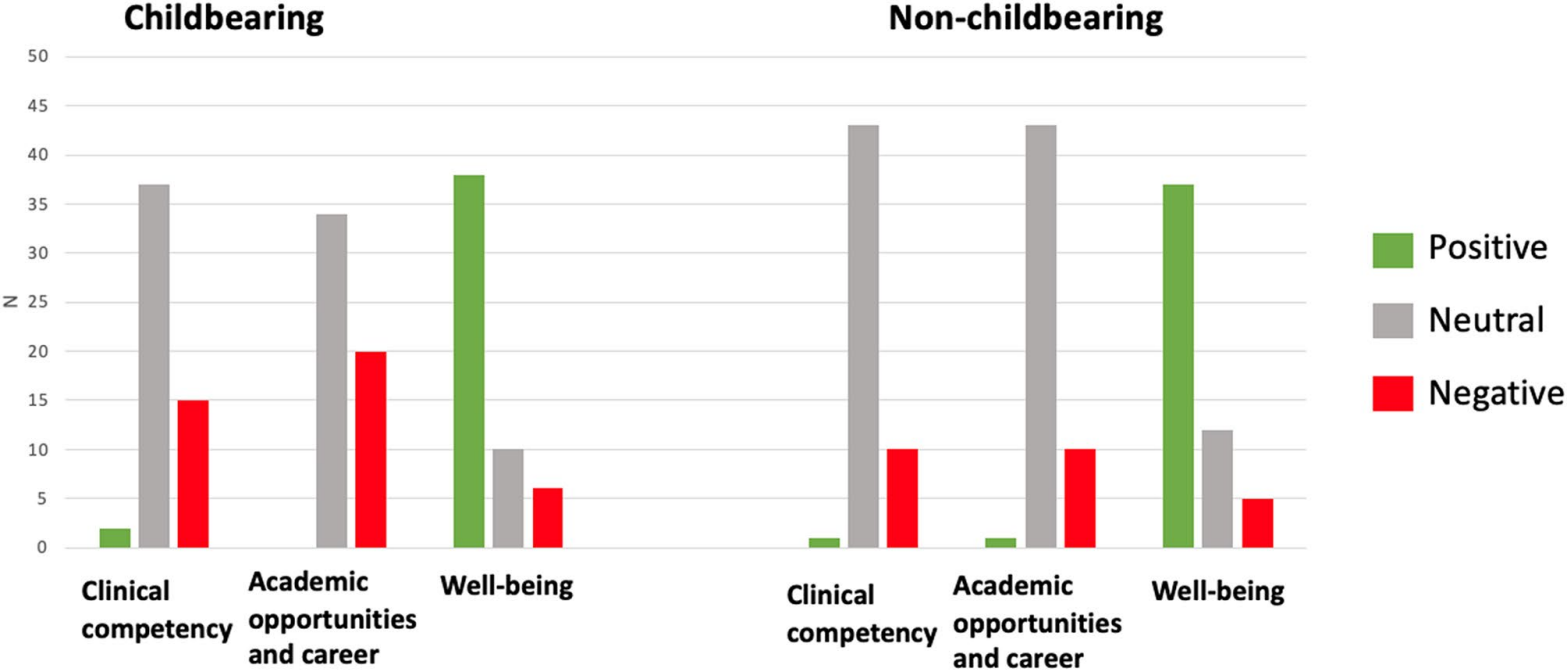
Perceived impact of parental leave on childbearing and non-childbearing residents Bar graphs show PDs’ perceptions of the impact of parental leave on resident clinical competency, academic opportunities and career development, and well-being. Number of PDs is shown on the y-axis. A positive impact is shown in green, neutral impact is shown in gray, and negative impact is shown in red

The majority of PDs perceived that parental leave had a positive impact on resident wellness for both childbearing (70%) and non-childbearing residents (69%). Most responded that they perceived that parental leave had a neutral impact on clinical competency, academic opportunities, and career development for both childbearing and non-childbearing residents (Fig. 3). There were no statistical associations between these perceptions and program size, maximum length of leave, program location, percentage of females in the program, and average number of leaves per year.

### Perceived barriers

**Fig. 4:**
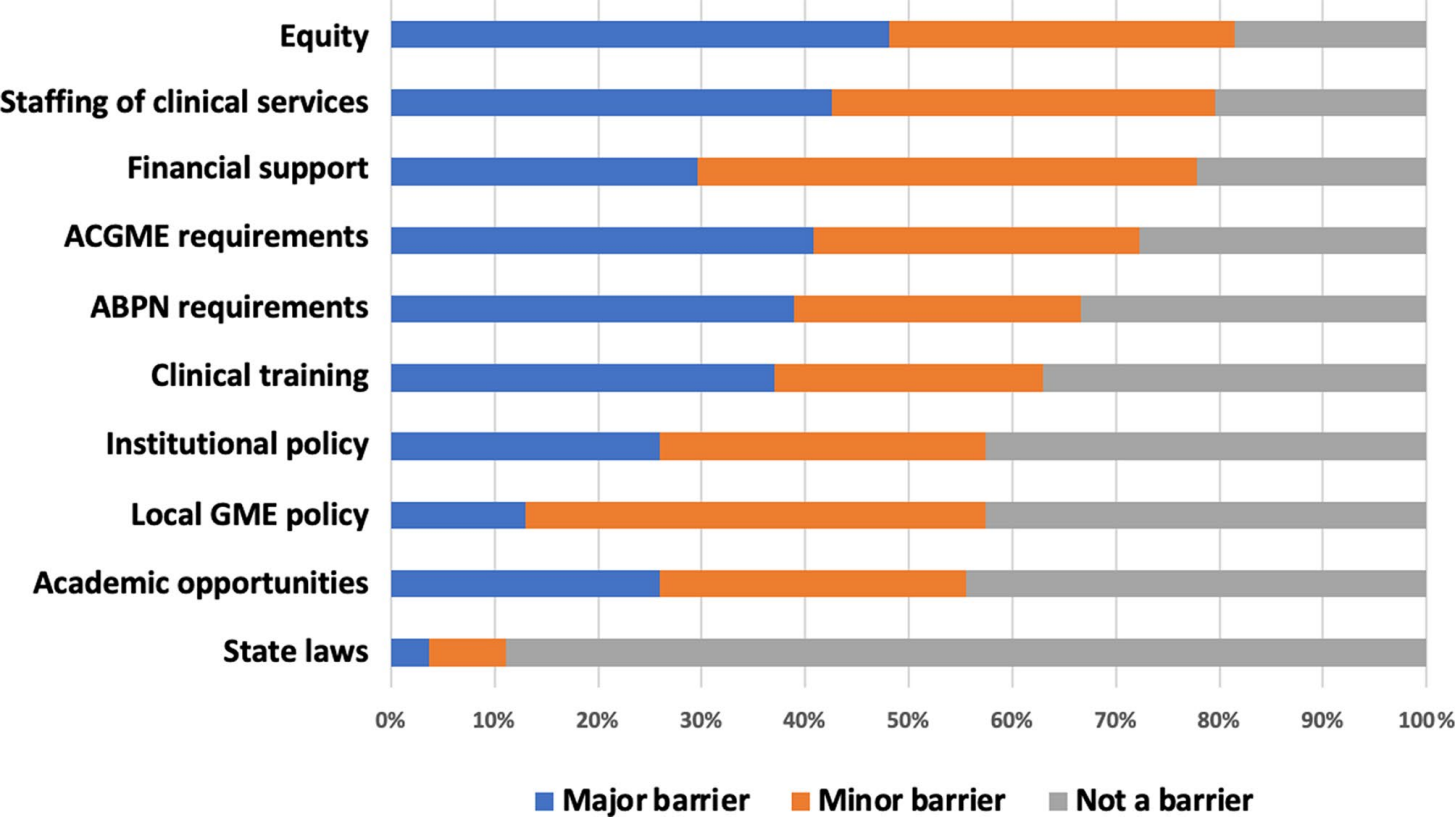
PDs identify major and minor barriers to augmenting parental leave policies. Barriers are separated in different colors by major vs. minor vs. not a barrier. Percentage of PDs is shown on the x-axis

The most common major and minor barriers to providing a 12-week paid policy were concerns about equity in the program and impact on other trainees (82%), staffing of clinical services (80%), and impact on clinical training (78%) (Fig. 4).

We assessed the impact of program size, maximum length of leave, program location, percentage of females in the program, and average number of leaves per year on each barrier. Programs with longer lengths of leave were less likely to find ACGME requirements a barrier (OR = 0.564 [0.407–0.782] for childbearing resident leave and 0.773 [0.632–0.946] for non-childbearing resident leave). Similarly, programs with longer lengths of leave were less likely to find ABPN requirements a barrier (OR = 0.666 [0.516–0.859] for childbearing residents and 0.803 [0.663–0.971] for non-childbearing residents). Programs with longer lengths of childbearing leave were also less likely to find local GME policy a barrier (OR = 0.805 [0.658–0.985]). This association was not seen for non-childbearing leave (OR = 0.990 [0.838–1.170]).

## Discussion

In our study surveying neurology PDs on parental leave policies, 87% responded that they have policies for both childbearing and non-childbearing residents, 7% have policies for childbearing residents only, and 6% have no policy or were not sure if they have a policy. Of the programs with parental leave policies for both childbearing and non-childbearing residents, 64% have the same policy for childbearing and non-childbearing residents. These results are comparable to a study which surveyed internal medicine PDs in 2019 and found that 82% of programs had policies for childbearing leave and 69% had policies for non-childbearing leave [16]. A systematic review of parental leave in GME found that formal leave policies in graduate medical education have varied over time and ranged from 22% of programs [17] in 1986 to 90% more recently [18]. This increase is encouraging, but there is still room for improvement. All residency programs should have clearly written policies for both childbearing and non-childbearing residents. To promote equity, programs should also strive to have robust policies for both childbearing and non-childbearing residents.

The average maximal length of leave allowed without extension of training was 8.5 weeks (range 0–13) for childbearing residents and 6.2 (range 0–13) for non-childbearing residents of the programs surveyed. There was no association between program size and length of leave. Though these averages are in line with the minimum 6 weeks of leave as required by the ABPN and ACGME, they fall below the goal of a 12 week leave that is recommended by the Family Medical Leave Act [19] and the American Medical Association (AMA) [5]. For childbearing parents, 12 weeks or more of maternity leave is associated with numerous benefits including lower rates of postpartum depression, improved maternal mental health [20], and duration of breastfeeding [21]. Leave for non-childbearing parents is also important for promoting parent-child bonding [22, 23], and improving health outcomes for children [24]. 

Most programs did not require full use of vacation or elective time, and 42% had policies for reducing clinical rotations to create a leave. Constructing leaves in this manner is an important strategy to promote equity and wellness. It is critical for programs to develop policies that have a balanced approach toward constructing leave, without using vacation time, and using reductions of both elective time and some clinical rotations where possible.

Importantly, most PDs perceived that parental leave had a positive impact on resident wellness for both childbearing (70%) and non-childbearing residents (69%). A multi-center study which surveyed female residents found that increased duration of parental leave had a positive impact on resident wellness [25]. Improving resident wellness is an important reason in favor of further increasing the duration of parental leave in neurology residency. Most PDs responded that parental leave had a neutral impact on clinical competency, academic opportunities, and career development for both childbearing and non-childbearing residents. This contrasts with negative perceptions of parenthood in residency which have previously been reported^18^ and dispels common reasons that may be cited to not offer longer parental leave. Interestingly, there were no statistical associations (within the limitations of our study) between these perceptions and program size, maximum length of leave, program location, percentage of females in the program, and average number of leaves per year.

The most common major and minor barriers cited by PDs to providing a 12-week paid policy were concerns about equity in the program and impact on other trainees (82%) and staffing of clinical services (80%). Parental leave policies can be crafted in ways that mitigate potential inequities among other trainees in the program, and institutional financial backing can support additional solutions for staffing of clinical services. We recently reported our experience with the development of a 12-week paid parental leave policy in the Mass General Brigham neurology residency. In our article we highlight strategies for programs to minimize the impact of a parental leave on other trainees, including use of APPs and making intentional upfront scheduling changes [26]. Only 10% of programs in our survey reported that APPs participate in clinical coverage for a parental leave. Though there are financial constraints to utilizing APPs to fill in for a leave, they are a valuable and underutilized resource to minimize schedule disruptions and impact on other residents.

Impact on clinical training was another common barrier to providing a 12-week paid parental leave policy. We did not specifically include clinical competency metrics in this study, but data in other specialties suggest that overall parental leave does not have a measurable impact on clinical training. A study of physical medicine and rehabilitation residents showed no difference in board certification exam passing rates among individuals who took a medical or parental leave [27]. A study of ophthalmology residents who took parental leave found no differences in average scores on standardized assessments, research activity, ACGME milestone scores, or surgical volumes [28]. Additional studies in neurology residency are needed to understand if time away from neurology residency objectively impacts clinical training.

We found that programs with longer lengths of leave were less likely to consider ACGME, ABPN, or local GME requirements a barrier. Though these results are not surprising, they highlight the importance of educating PDs on these policies.

Our study has several limitations. Potential recall bias and a relatively low response rate may have skewed the results of the survey. It is also possible that those PDs who responded were more invested in parental leave policies and the results may be favorably biased. While we asked PDs about their perception of parental leave on wellness, academic opportunities, and career development, we did not look at objective measurements such as board examination scores, or clinical assessments. Because we did not obtain data on when leaves were taken in relation to completion of the survey, this study may also be limited by recency bias. PDs who had residents that took a parental leave years ago may have different recollections about the leave compared to PDs who had residents who took a more recent leave. Despite these limitations, we believe our study provides important insights on the current landscape of parental leave policies in neurology residency. These findings have value as more programs prioritize this issue and seek ways to augment parental leave.

In conclusion, there was variability in length and composition of leave among neurology residency programs. Overall PDs felt parental leave had a neutral impact on clinical competency, and academic opportunities, and a positive impact on well-being. Policies to improve parental leave should focus on addressing commonly perceived barriers, such as staffing of clinical services, and minimizing scheduling disruptions to promote equity among residents.

### Electronic supplementary material

Below is the link to the electronic supplementary material.

Supplementary Material 1

## Data Availability

The datasets used and/or analysed during the current study are available from the corresponding author on reasonable request.
