# Supplementary material for: Barriers to increasing paid parental leave in U.S. neurology residencies: a survey of program directors
Source: BMC Med Educ. 2024 Apr 9;24:387. doi: 10.1186/s12909-024-05333-1 (PMC11003009; doi:10.1186/s12909-024-05333-1)
Supplement: Supplementary file 1 — Supplementary Material 1 [file 12909_2024_5333_MOESM1_ESM.pdf]

Neurology Residency Parental Leave Survey

Does your program have an established parental leave policy for residents?

◯ Yes, for both childbearing and non-childbearing residents
◯ Yes, for childbearing residents only
◯ No, for neither childbearing or non-childbearing residents
◯ I'm not sure

Is the policy the same for childbearing and non-childbearing parents?

◯ Yes
◯ No
◯ I'm not sure

How many years ago was your parental leave policy last updated?

◯ Within the past year
◯ Two to five years years ago
◯ Five to ten years ago
◯ Greater than ten years ago
◯ I'm not sure

Are you currently in the process of revising your parental leave policy?

◯ Yes
◯ No

Are you currently in the process of creating a parental leave policy?

◯ Yes
◯ No

What is the maximum length of parental leave that an individual can take in your program without extension of training assuming there are no concerns regarding their clinical competency

For a childbearing resident?

◯ 0 weeks
◯ 1 week
◯ 2 weeks
◯ 3 weeks
◯ 4 weeks
◯ 5 weeks
◯ 6 weeks
◯ 7 weeks
◯ 8 weeks
◯ 9 weeks
◯ 10 weeks
◯ 11 weeks
◯ 12 weeks
◯ more than 12 weeks
◯ I'm not sure

For a non-childbearing resident?

◯ 0 weeks
◯ 1 week
◯ 2 weeks
◯ 3 weeks
◯ 4 weeks
◯ 5 weeks
◯ 6 weeks
◯ 7 weeks
◯ 8 weeks
◯ 9 weeks
◯ 10 weeks
◯ 11 weeks
◯ 12 weeks
◯ more than 12 weeks
◯ I'm not sure

Under your policy does a trainee receive full pay and benefits during their parental leave?

◯ yes, for the entire parental leave
◯ yes, for a portion of the parental leave, and reduced pay for the remainder
◯ no, reduced pay throughout the parental leave
◯ I'm not sure

Does your policy require use of vacation time to create a parental leave?

◯ No
◯ Yes, part of the vacation time must be used
◯ Yes, all of the vacation time must be used
◯ I'm not sure

Does your policy require use of elective time to create a parental leave?

◯ No
◯ Yes, some of the elective time must be used
◯ Yes, all of the elective time must be used
◯ I'm not sure

Does your policy allow for reduction in clinical rotations to create a parental leave?

◯ No
◯ Yes
◯ I'm not sure

Are residents required to make up call they miss as part of their leave?

◯ No
◯ Yes, some of the scheduled calls
◯ Yes, all of the scheduled calls
◯ I'm not sure

Who is responsible for scheduling a parental leave (check all that apply)?

☐ The trainee taking the leave
☐ Chief residents
☐ Program Director
☐ Other

If other, indicate who:

____

Who covers for the resident taking leave (check all that apply)?

☐ Other neurology residents
☐ Fellows
☐ Advanced practice providers
☐ Attending physicians

A recent NEJM editorial recommended that medical trainees receive 12 weeks of parental leave during residency. What do you consider to be barriers to providing a 12 week paid parental leave at your institution?

	Major barrier	Minor barrier	Not a barrier
State laws	◯	◯	◯
ACGME residency accreditation requirements	◯	◯	◯
ABPN board eligibility requirements	◯	◯	◯
Institutional policy	◯	◯	◯
Local GME policy	◯	◯	◯
Staffing of clinical services	◯	◯	◯
Financial support	◯	◯	◯
Concerns about clinical training	◯	◯	◯
Concerns about academic opportunities and career development	◯	◯	◯
Concerns about equity in the program and impact on other trainees	◯	◯	◯

How long have you been program director or associate program director?

◯ 1 year
◯ 2 years
◯ 3 years
◯ 4 years
◯ 5 years
◯ 6 years
◯ 7 years
◯ 8 years
◯ 9 years
◯ 10 years
◯ more than 10 years

Where is your program located?

◯ Northeast
◯ Midwest
◯ West
◯ Southwest
◯ Southeast

How many adult neurology residents per year are currently in your program?

◯ 1
◯ 2
◯ 3
◯ 4
◯ 5
◯ 6
◯ 7
◯ 8
◯ 9
◯ 10
◯ 11
◯ 12
◯ 13
◯ 14
◯ 15
◯ 16
◯ 17
◯ 18
◯ 19
◯ 20
◯ 21
◯ 22
◯ 23
◯ 24
◯ 25

What is the percentage of female residents currently in your program?

◯ < 20%
◯ 20% to < 40%
◯ 40 to < 60%
◯ 60 to < 80%
◯ 80 to 100%

Approximately how many parental leaves per year were scheduled in your program over the past several years

For childbearing residents?

◯ no leaves per year
◯ between zero and one leave per year
◯ one leave per year
◯ two leaves year
◯ three leaves per year
◯ four leaves per year
◯ five leaves per year
◯ more than five leaves per year

For non-childbearing residents?

◯ no leaves per year
◯ between zero and one leave per year
◯ one leave per year
◯ two leaves year
◯ three leaves per year
◯ four leaves per year
◯ five leaves per year
◯ more than five leaves per year

For childbearing residents, how much do you think parental leave impacts the following aspects of training?

	Significantly negative impact	Somewhat negative impact	No impact/neutral	Somewhat positive impact	Very positive impact
Clinical competency	◯	◯	◯	◯	◯
Academic opportunities and career development	◯	◯	◯	◯	◯
Well-being	◯	◯	◯	◯	◯

For non-childbearing residents, how much do you think parental leave impacts the following aspects of training?

	Significantly negative impact	Somewhat negative impact	No impact/neutral	Somewhat positive impact	Very positive impact
Clinical competency	◯	◯	◯	◯	◯
Academic opportunities and career development	◯	◯	◯	◯	◯
Well-being	◯	◯	◯	◯	◯

Please feel free to provide any other ideas about your experience with parental leave in neurology residency.
